# Sustainability in Internal Medicine: A Year-Long Ward-Wide Observational Study

**DOI:** 10.3390/jpm14010115

**Published:** 2024-01-20

**Authors:** Giuseppe A. Ramirez, Sarah Damanti, Pier Francesco Caruso, Francesca Mette, Gaia Pagliula, Adriana Cariddi, Silvia Sartorelli, Elisabetta Falbo, Raffaella Scotti, Gaetano Di Terlizzi, Lorenzo Dagna, Luisa Praderio, Maria Grazia Sabbadini, Enrica P. Bozzolo, Moreno Tresoldi

**Affiliations:** 1 Unit of Immunology, Rheumatology, Allergy and Rare Diseases, IRCCS Ospedale San Raffaele, 20132 Milan, Italy; 2 Faculty of Medicine, Università Vita-Salute San Raffaele, 20132 Milan, Italyelisabetta.falbomd@gmail.com (E.F.);; 3 Unit of General Medicine and Advanced Care, IRCCS Ospedale San Raffaele, 20132 Milan, Italyditerlizzi.gaetano@hsr.it (G.D.T.); tresoldi.moreno@hsr.it (M.T.)

**Keywords:** sustainability, general internal medicine, healthcare resources, hospital-acquired infections, length of stay, in-hospital mortality

## Abstract

Population aging and multimorbidity challenge health system sustainability, but the role of assistance-related variables rather than individual pathophysiological factors in determining patient outcomes is unclear. To identify assistance-related determinants of sustainable hospital healthcare, all patients hospitalised in an Internal Medicine Unit (n = 1073) were enrolled in a prospective year-long observational study and split 2:1 into a training (n = 726) and a validation subset (n = 347). Demographics, comorbidities, provenance setting, estimates of complexity (cumulative illness rating scale, CIRS: total, comorbidity, CIRS-CI, and severity, CIRS-SI subscores) and intensity of care (nine equivalents of manpower score, NEMS) were analysed at individual and Unit levels along with variations in healthcare personnel as determinants of in-hospital mortality, length of stay and nosocomial infections. Advanced age, higher CIRS-SI, end-stage cancer, and the absence of immune-mediated diseases were correlated with higher mortality. Admission from nursing homes or intensive care units, dependency on activity of daily living, community- or hospital-acquired infections, oxygen support and the number of exits from the Unit along with patient/physician ratios were associated with prolonged hospitalisations. Upper gastrointestinal tract disorders, advanced age and higher CIRS-SI were associated with nosocomial infections. In addition to demographic variables and multimorbidity, physician number and assistance context affect hospitalisation outcomes and healthcare sustainability.

## 1. Introduction

Admissions to Internal Medicine departments constitute the bulk of hospital workload [1,2] and diseases lying in the broad set of Internal Medicine disorders contribute to the largest share of healthcare expenditures [3]. Patients admitted to Internal Medicine departments are more often characterised by multimorbidity. Increased prevalence of multimorbidity and growing patient complexity, in turn, are direct consequences of the progressive extension of the lifespan in the general population [4]. An aging population constitutes a major social issue due to the increasing healthcare demand and concomitant reducing workforce to produce adequate economic support to potentially increased health-related costs [5]. This growing disequilibrium, in turn, challenges the stability of organisational models such as the United Kingdom or the Italian National Health Systems, which were developed in the XX century under radically different demographic conditions [6]. Therefore, innovative solutions at the political and administrative level are needed to preserve and possibly improve the quality of healthcare services in a sustainable way [7].

Sustainability can be generally defined as the ability to collect and employ sufficient resources to avoid resource exhaustion or prevent the capacity to sustain a given task from becoming irreversibly degraded [8]. In the setting of economics and social sciences, the term is usually intended according to the definition by Brundtland et al. as “meeting the needs of the present without compromising the ability of future generations to meet their own needs” [9]. In countries endowed with public welfare policies, this concept has generally been translated into the need to fit expected healthcare expenditures with available funding. This perspective possibly downsizes the multifaceted matter of sustainability to financial aspects only [10]. Considering healthcare-related financial costs as the only determinants of sustainability might fail to take into account the more complex economic costs of poor health. In fact, these might include additional expenditures to address complicated or delayed health problems, which, conversely, can be prevented by programmes of early intervention/screening in the community or by policies aiming at minimising hospital-related adverse events such as nosocomial infections [10,11]. Furthermore, the idea that reducing healthcare-related costs can coexist with stable or improving qualitative standards relies on the assumption that healthcare workers’ resilience is sufficient to address increasing workloads due to growing patient complexity amidst reduced resources. However, little is known about the potential boundaries of this assumption, that is whether reduced healthcare resources already have a measurable, detrimental impact on patient outcomes, especially in hospital settings [12,13]. More generally, there is limited evidence on the specific role of assistance-related variables in affecting the course of hospitalised patients, with most studies focusing on individual pathophysiological characteristics of clinically relevant events.

To address this issue, we designed a prospective year-long ward-wide study aiming to measure the burden of adverse hospitalisation outcomes in an Internal Medicine Unit from a large university hospital and to dissect the role of individual risk factors from assistance-related variables.

## 2. Materials and Methods

### 2.1. Enrolment and Study Timeframe

Upon informed consent, all patients admitted to the Unit of General Medicine and Advanced Care at IRCCS Ospedale San Raffaele, Milan, Italy starting 15th February 2016 were consecutively enrolled in a prospective observational study (the SIM, “Sostenibilità In Medicina”, protocol), conforming to the Declaration of Helsinki and approved by the Institutional Review Board of the same Institution (reference number 110/INT/2015). At time of data collection, the Unit of General Medicine and Advanced Care was a 43-bed Internal Medicine Department dedicated to complex acute inpatients in the setting of a large University Hospital. The start-up phase was continued until the end of February 2016, when data from 43/43 beds were available in parallel. Patient enrolment was continued until March 2017, that is 365 days after the end of the start-up phase. Data collection ended after discharge of the last enrolled patient in July 2017 (Figure S1A). The main inclusion criteria consisted of being admitted to the Unit of General Medicine and Advanced Care and consenting to data collection and analysis. There were no exclusion criteria.

### 2.2. Assessment of Patient Complexity and Intensity of Care

Patient data, including demographics (age, sex), clinical features at presentation and during the hospitalisation course, along with measures of intensity of care, were collected on a daily basis from each subject (Figures S1 and S2). Cardiac, vascular/haematological, endocrine/metabolic, respiratory, upper and lower gastrointestinal, hepatic, renal, genitourinary, musculoskeletal or cutaneous, ophthalmologic or ear-nose-throat, psychiatric and neurological morbidity along with hypertension were graded on a 0–4 discrete scale according to the Cumulative Illness Rating Scale (CIRS) algorithm [14]. Besides the total CIRS score, we calculated the CIRS-associated severity index (CIRS-SI) in its original [14] and revised formulations [15], along with the CIRS comorbidity index (CIRS-CI). Each CIRS component was also recorded as a dichotomous variable with any value above zero being counted as positive. Additional binary comorbidity variables included the absence vs. presence of immune-mediated disorders, infections and cancer. An arbitrary 0–4 scale was also calculated for cancer prognosis (>10 years, 5–10 years, >6 months, less than six months). Furthermore, we recorded whether patients were immunocompromised (either by concomitant disorders or treatments), whether they required any surgical intervention and whether they were dependent in their activities of daily living (ADL) or instrumental ADL (IADL; Figure S2). Patient assessment was performed by senior physicians and residents, who were specifically trained on the study procedures.

Data regarding the use of oxygen supplementation through nasal cannulas or Venturi’s mask, non-invasive mechanical ventilation (NIMV), continuous monitoring of vital signs (including fluid balance), intravenous treatments, treatment with one or more vasoactive drugs and renal replacement treatments were also collected daily. In addition, we recorded any exit from the ward for diagnostic or treatment procedures and the need for non-routine in-ward procedures. These binary data were used to estimate patient intensity of care through the nine equivalent of nursing manpower score (NEMS; Figure S2) [16].

### 2.3. Assistance Parameters

The number of senior physicians along with the number of nurses actively employed in patient assistance was calculated on a daily basis. In addition, we recorded whether each patient had accessed the Unit from the Emergency Department or from environments at risk for nosocomial infections such as nursing homes or intensive care units.

### 2.4. Individual and Global Ward Data Aggregation

Variations in qualitative and quantitative variables throughout individual hospitalisation courses were assessed at an individual and ward-wide level and summarised by calculating individual and global ward mean and worst value (the highest or lowest values depending on the variable characteristics). Derived continuous and binary variables were rounded to the nearest integer (Figure S1B).

### 2.5. Outcomes

We defined in-hospital death vs. discharge/transfer to other units and nosocomial infections as the main categorical outcomes of interest (Figure S2). In addition, we used the length of hospital stay in survivors as a proxy of patient care efficiency. Nosocomial infections were defined as fungal, bacterial or viral infectious events occurring after 48 h from admission. Patients deceased or transferred to other Units with non-resolved infections on admission were excluded from nosocomial infection statistics. Patients with nosocomial infections having been acquired in other Units before admission and without temporal details on infection onset were also excluded from nosocomial infection statistics.

### 2.6. Statistical Analysis

Data digitalisation was performed through a dedicated in-house software based on Microsoft Excel^®^ 2013 and modelled on a similar software for clinical data collection [17]. After data collection, enrolled patients were randomly subdivided 2:1 into a training subset and a validation subset by using the “RANDBETWEEN” function of Microsoft Excel. Descriptive statistics were performed on the whole patient population. Univariate and multivariate outcome analyses were first performed in the training subset and validated in the validation subset. Statacorp STATA version 15.0 was used for statistical analyses. *p*-values below 0.05 were considered significant.

The chi-square test was used to compare categorical variables among groups. Shapiro–Wilk tests were performed on quantitative variables to test for normal distribution. Mann–Whitney and Kruskal–Wallis tests were employed for univariate analyses of differential trends in non-normally distributed quantitative time-independent variables among two or more groups, respectively. Student *t*-test and ANOVA were used for the same purpose with normally distributed variables. Bivariate correlation among quantitative variables was performed through the Spearman’s or Pearson’s test, as appropriate. Sample size calculations for univariate tests for the hypothesis of a differential length of stay among patients with vs. without selected clinical or assistance-related characteristics [18,19] were performed by setting significance alpha level to 0.05 and target power to 0.80. We estimated the need for more than 460–580 study patients with these parameters.

Univariate Cox’s regression analyses were used to identify potential associations of categorical and/or quantitative variables with death and nosocomial infections. Sample size calculations to test the hypothesis that clinical or assistance-related variables could affect these outcomes were performed by setting the significance alpha level to 0.05 and target power to 0.80 [20]. Based on previous evidence [21,22], we estimated a required sample size of at least 220–480 subjects (with an estimated event rate of 10%) for overall survival analyses. Similarly, at least 220–540 subjects (with an estimated event rate of 15%) were estimated to be needed for nosocomial infection analyses [23,24].

Classification and regression trees were used to assess the combined contribution of multiple categorical and quantitative variables to these time-dependent binary outcomes. The minimum size of each branch was set to 25 subjects. Hazard ratios generated through these algorithms in the training subset were log-transformed, rounded to the nearest integer and employed to classify patients in the validation subset according to their expected risk of incurring in death or nosocomial infection. Cox’s regression analyses were then performed to challenge the ability of expected risk estimates to efficiently predict the actual occurrence of the outcomes of interest.

A generalised linear model was built to identify potential quantitative and categorical predictors of the length of hospitalisation. The test was set assuming gamma distribution and using identity as the link function. Correlation coefficients and constant terms obtained in the training subset through the generalised linear model were employed to obtain predicted lengths of stay in the validation subset. Expected lengths of stay generated through this procedure were compared to real measurements by Spearman’s bivariate correlation. Redundant variables were excluded from multivariate analyses.

Data are presented as median (interquartile range, IQR) for quantitative variables and percentage for categorical variables, unless otherwise specified.

## 3. Results

### 3.1. General Clinical Features and Outcomes

Of 1173 enrolled subjects, 1073 entered the study in the 365-day timeframe (Figure S1) and were considered for analysis. Most patients were males (59%) and the median (IQR) age was 74 (62–82) years. Cardiovascular (69%) and pulmonary disorders (48%) constituted the most frequent causes of morbidity. In addition, 436 subjects (41%) were dependent on assistance for ADL/IADL. The median (IQR) NEMS was 18 (16–19). Consistently, more than half of the patients required oxygen support for the majority of their hospitalisation and >70% required exiting from the ward for diagnostic or therapeutic procedures. More than two-thirds of patients (735/1073, 68%) had an infection on admission. Infections on admissions were more frequent in patients admitted from high-risk environments for healthcare-associated infections such as intensive care units and nursing homes (85/105, 81%) than in non-institutionalised patients (650/968, 67%, χ^2^ = 8.365; *p* = 0.004). In addition, 136 (13%) patients developed an infection during hospitalisation. There were 119 in-hospital deaths. Surviving patients were discharged after a median of 12 (8–20) days from admission. Additional patient descriptives disaggregated by sex are reported in Table 1.

The median (IQR) patient/physician ratio was 8 (7–9) and 18 (17–20) by excluding or not bank holidays, respectively. The patient/nurse ratio was 7 (7–7). The median discharge rate was 2/43 patients/day (IQR = 0–3). The daily number of discharged patients was inversely correlated with the corresponding patient/physician ratio (rho = −0.292; *p* < 0.001).

### 3.2. Mortality

There were 81/726 and 38/347 deaths in the training and validation subsets, respectively. Univariate Cox regression analysis in the training subset showed that in-hospital mortality increased with age (HR = 1.04, 95% CI = 1.02–1.07, *p* < 0.001) and was significantly associated with ADL/IADL dependence (HR = 1.82, 95% CI = 1.16–2.87, *p* = 0.010). A higher comorbidity burden (total CIRS, CIRS-SI and CIRS-CI) correlated with a higher risk of mortality (*p* < 0.001 for all three variables). Cardiovascular disorders (HR 1.83, 95% CI = 1.07–3.13, *p* = 0.027) including hypertension (HR = 1.73, 95% CI = 1.09–2.73; *p* = 0.019) and end-stage cancer (HR = 2.31, 95% CI = 1.25–4.27, *p* = 0.008) were negatively associated to survival. Immune-mediated disorders (HR = 0.38, 95% CI 0.17–0.83, *p* = 0.016) and admittance from nursing homes or ICUs (HR = 0.31, 95% CI = 0.14–0.70, *p* = 0.005) were associated with a lower risk of death. Patients showing a higher NEMS (HR 1.06, 95% CI = 1.02–1.12, *p* = 0.007) and requiring circulation support with at least one drug (HR = 8.97, 95% CI= 4.09–16.69) had a higher risk of death, while increasing number of exits from the Unit were associated with higher survival rates (HR = 0.82, 95%CI 0.74–0.90, *p* < 0.001; Table S1).

**Figure 1:**
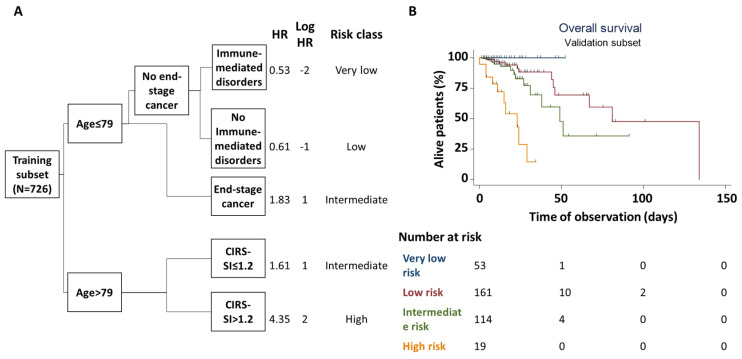
Multivariate derivation and validation of factors associated with in-hospital mortality. (**A**) depicts the classification and regression tree analysis for in-hospital mortality in the training subset. The model included age, ADL/IADL dependency, cardiovascular disorders, immune–mediated disorders, end-stage cancer, admission from nursing homes or ICUs, average cumulative illness rating scale severity index (CIRS-SI), average nine equivalents of manpower score (NEMS), circulation support with at least one vasoactive drug. Only age, CIRS-SI, end-stage cancer and (absence of) immunological disorders were identified as significant strata for patient risk classification. Based on derived, log-transformed hazard ratios (HR), four risk groups were defined (very low risk, blue; low risk, red; intermediate risk, green; high risk, orange). This classification was then applied to patients in the validation subset (**B**) to assess its performance in identifying patients at higher vs. lower risk of in-hospital death. Cox’s regression analysis in this subset confirmed the significant correlation between the derived risk classification and the actual risk of in-hospital mortality (Log rank = 24.91; *p* < 0.001).

Classification and regression tree analysis in the training subset identified age, CIRS-SI, immune-mediated disorders and end-stage cancer as the main determinants of patient in-hospital survival. Specifically, patients with age > 79 years and CIRS-SI > 1.2 had the highest risk of death, while patients with age ≤ 79, immune-mediated disorders and no end-stage cancer had the lowest risk. Patients with age > 79 and CIRS-SI ≤ 1.12 or with age ≤ 79 and end-stage cancer had an intermediate risk of in-hospital death (Figure 1A). Risk groups identified in the training subset were able to efficiently stratify patients in the validation subset according to their actual survival profile (Figure 1B)

### 3.3. Length of Hospitalisation

Factors associating with hospitalisation length were analysed in 645 survivors in the training subset and 309 survivors in the validation subset. In the training subset, patients with ADL/IADL dependence, neurological disorders and/or infections had longer hospitalisation compared to patients without these clinical characteristics. Nosocomial infections were strongly associated with prolonged hospitalisations. Assistance-related variables affecting the individual patient course such as admission from nursing homes or ICUs, need for surgical intervention(s) and for oxygen support with and without NIMV were associated with longer stays in the ward (Table 2). The number of exits from the Unit (rho = 0.622, *p* < 0.001) and of non-standard in-ward procedures (rho = 0.272, *p* < 0.001) along with the average individual NEMS (rho = 0.173, *p* < 0.001) were also significantly correlated with the duration of hospitalisation. In addition to individual variables, Unit-related parameters such as the average length of hospitalisation (rho = 0.079, *p* = 0.046), the number of discharged patients (rho = −0.138, *p* = 0.001), the prevalence of infected subjects (rho = 0.087, *p* = 0.027) and of NIMV users (rho = 0.117, *p* = 0.003) during each patient course were significantly correlated with their respective length of stay. The average patient/physician ratio also directly correlated with hospitalisation length (rho = 0.117, *p* = 0.003; Table S2).

At multivariate analysis, admission from high-risk settings such as nursing homes and ICUs, ADL/IADL dependence, community- or hospital-acquired infections, need for oxygen support and the number of exits from the Unit affected hospitalisation length. A higher average patient/physician ratio was also independently associated with longer hospitalisations (Table 3). Predicted hospitalisation lengths in the validation subset based on analyses in the training subset were strongly correlated with real measurements (rho = 0.678; *p* < 0.001; Figure S3). The median (IQR) discrepancy between expected and measured values was ±3.5 (1.5–7.5) days.

### 3.4. Nosocomial Infections

A total of 681/726 patients in the training subset and 324/347 patients in the validation subset were included for nosocomial infection analyses, with 92/681 and 43/324 having at least one nosocomial infection. Univariate analysis in the training subset showed that patients of older age were at increased risk of nosocomial infections (HR = 1.03, 95% CI = 1.02–1.05, *p* < 0.001). Higher clinical complexity as estimated by CIRS scores was also significantly associated with a higher likelihood of developing at least one hospital-acquired infection (Table S3). In particular, cardiovascular disorders (HR = 1.73, 95% CI 1.06–2.82, *p* = 0.029) including hypertension (HR = 1.56, 95% CI = 1.02–2.42, *p* = 0.041) and cardiac disorders (HR = 1.73, 95%CI = 1.13–2.66, *p* = 0.012) along with upper gastrointestinal tract diseases (HR = 2.59, 95% CI 1.54–4.35, *p* < 0.001) were all associated with higher rates of nosocomial infections. Patients with higher NEMS (HR = 1.06, 95%CI 1.01–1.11, *p* = 0.019), requiring oxygen (HR = 1.74, 95% CI = 1.10–2.74, *p* = 0.018), circulation support (HR = 4.67, 95% CI = 1.14–19.13, *p* = 0.032) and/or a higher number of non-standard in-ward procedures (HR = 1.61, 95% CI = 1.01–1.33, *p* = 0.032) had higher nosocomial infection rates compared to patients with lower intensity of care (Table S3).

**Figure 2:**
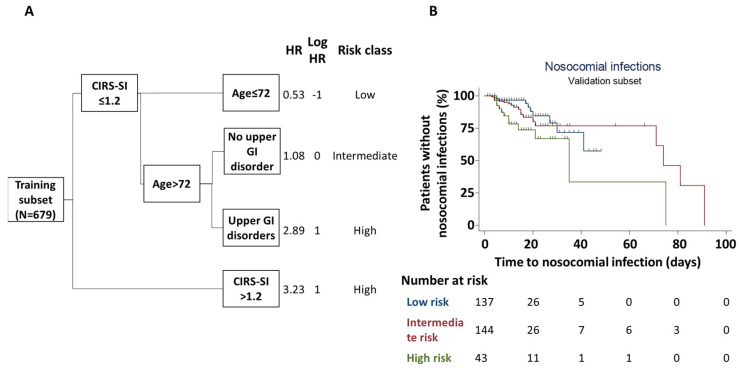
Multivariate derivation and validation of factors associated with nosocomial infections. (**A**) depicts the classification and regression tree analysis for the development of nosocomial infections in the training subset. The model included age, cardiovascular disorders, upper gastrointestinal (GI) disorders, average cumulative illness rating scale severity index (CIRS–SI), average nine equivalents of manpower score (NEMS), use of any form of oxygen support, circulation support with at least one vasoactive drug and total number of in-ward non-standard procedures. Only age, CIRS–SI and upper GI disorders were identified as significant strata for patient risk classification. Based on derived, log-transformed hazard ratios (HR), three risk groups were defined (low risk, blue; intermediate risk, red; high risk, green). This classification was then applied to patients in the validation subset (**B**) to assess its performance in identifying patients at higher vs. lower risk of nosocomial infection. Cox’s regression analysis in this subset confirmed the significant correlation between the derived risk classification and the actual risk of nosocomial infection (Log rank = 5.38; *p* = 0.020).

Multivariate classification and regression tree analysis in the training subset identified age, CIRS-SI and upper gastrointestinal tract disorders as the main factors determining the risk of hospital acquired infections. Patients with CIRS-SI ≤ 1.2 and age ≤ 72 were protected from the development of nosocomial infections while patients with CIRS-SI > 1.2 or age > 72 and upper gastrointestinal tract disorders had the highest risk. Patients of more than 72 years of age without upper gastrointestinal tract disorders had an intermediate risk of acquiring an infection during their stay in hospital (Figure 2A). When patients in the validation subset were stratified according to these parameters, we observed a significant association between risk classification and actual rates of observed nosocomial infections (Log rank = 5.38; *p* = 0.020; Figure 2B).

## 4. Discussion

We performed a comprehensive daily collection of individual and ward average data on clinical and assistance parameters to identify potential factors associated with mortality, prolonged hospitalisation and occurrence of nosocomial infections in an Internal Medicine Unit. In order to maximise data representativeness, we consecutively enrolled the whole population of patients admitted in the Unit over one year. We found that older age and a higher burden of active comorbidities were associated with reduced survival and increased likelihood to acquire in-hospital infections. In turn, nosocomial infections severely affected the length of hospitalisation. The linkage among active comorbidities (high CIRS-SI scores), nosocomial infections and in-hospital deaths indicates that patient general complexity is a major unaddressed driver of poor hospitalisation outcomes. Our results show that assistance-related factors, such as the number of physicians involved in patient care, are also associated with distinct patient outcomes, possibly suggesting that the detrimental impact of non-modifiable traits (such as age or chronic disorders) may be sustainably managed by workforce potentiation, especially in case of higher intensity of care [19,25]. On the other hand, we found that distinct adverse outcomes, such as death, might be associated with selected conditions, such as cancer or diseases other than immune-mediated disorders, while nosocomial infections might selectively cluster with upper gastrointestinal disorders. These data indicate that, besides quantitative changes in hospital workforce, improvements are also needed in current practice, possibly with the adoption of personalised strategies to address individual- or group-specific determinants of complexity and, eventually, of morbidity and mortality [26] through multidisciplinary teams [27,28,29]. In particular, patients with vulnerability factors for airborne and bloodborne infections such as those with impaired ability to protect their airways, receiving oxygen support or undergoing invasive procedures may require dedicated assistance paths to minimise the risks of nosocomial infections [26,30].

In our cohort, 68% of patients presented with an infection and 13% developed hospital-acquired infections, corroborating the notion that infectious diseases constitute a leading cause of morbidity in patients hospitalised in Internal Medicine Departments [31]. Nosocomial infection prevalence in our study was consistent with data from the same historical period and the same geographical area [30] and in line with current worsening trends compared to previous decades [32,33]. Nosocomial infections constitute a major cause of morbidity and increased healthcare costs [33,34,35] and affect the length of hospitalisation [18,36]. Disproportionately long hospitalisations, in turn, constitute a risk factor for nosocomial infections [30,37,38], suggesting that tackling factors associated with each of these two events may have a synergistic role on both of them.

Efficient cooperation among distinct nodes of the healthcare network, from hospital units to community care [39], might further contribute to minimise risk factors for nosocomial infections and reduce the length of hospitalisations, ultimately lowering health-related costs for preventable complications. Conversely, budget-oriented reforms of National Health Systems not taking into account these principles might exacerbate system vulnerabilities and undermine healthcare sustainability at hospital and non-hospital levels [6,12,40,41,42]. Consistently, we found that previous institutionalisation into ICUs or nursing homes was associated with both infection on admission and prolonged stay later on. Notably, although this study preceded the advent of severe acute respiratory syndrome coronavirus 2 (SARS-CoV-2)-related disease during the (COVID-19) pandemic, it highlighted crucial weaknesses of National Health Systems maladapting to changes in population demography and morbidity burden, which became dramatically evident during the first wave of the COVID-19 pandemic. In fact, during this recent crisis, those settings where the healthcare network was more fragmented and depowered became particularly susceptible to the uncontrolled spread of SARS-CoV-2, along with secondary infections [43], and suffered high mortality rates. This issue was particularly relevant in nursing homes [40,44] and was correlated to the degree of basal individual comorbidity [45]. Conversely, settings where programmes connecting hospitals to peripheral facilities (such as nursing homes) were implemented had lower COVID-19-related mortality rates [46].

Adding to the growing literature [47,48,49], our data may suggest multiple potential areas of intervention for a revision of current healthcare organisational models to face incumbent challenges for the next future, including further increases in healthcare demands due to population aging and sudden surges of acute illnesses during pandemics. Detecting a direct association between patient/physician ratio and hospitalisation length, along with the reciprocal relations among nosocomial infections, hospitalisation length and patient complexity, suggests that increasing staff number size proportionately to inflating health demand is fundamental to ensure the long-term sustainability of internal medicine hospital settings. This task is currently complicated by the growing severity of physician shortages worldwide [7,50]. Short-sighted countermeasures to this issue have historically included potentiating occasional shifts and repurposing of existing personnel [51]. However, these strategies might compromise continuity of care (besides personnel motivation and quality of care in areas becoming secondarily underpowered), which directly affects hospitalisation outcomes, including survival [19,52]. Conversely, increasing the number of physicians with specific professional skills in the management of patients with unprecedented complexity could constitute a more constructive way to address the problem of both higher healthcare demand and falling physician motivation to continue their career [51,53]. Transitioning from clinical decision support tools (guidelines, algorithms, disease classification criteria, clinimetrics) focused on single diseases to more comprehensive approaches to multi-morbidity scenarios and to individualised diagnostic-therapeutic paths might be part of this future professionalism [48,51]. Our data, showing that Unit-level variations in intensity of care and average patient complexity affect individual outcomes, also indicate the need for moving beyond a rigid approach to individuals detached from their Unit context and from the healthcare system continuum [46]. Taken together, these considerations point towards a paradigm shift from medicine of distinct diseases and of independent healthcare facilities to a more comprehensive idea of medicine of patient and assistance complexity [48,54]. Adaptation to fluctuating health demands and resource availability would be crucial in this setting to address future public health challenges, including the spread of new infectious agents [55]. Broad spectrum microbiological surveillance programmes, actively involving peripheral nodes in the healthcare system such as nursing homes have been proposed as cornerstone strategies for improved preparedness to new pandemics and to the spread of antibiotic resistant bacterial strains causing severe nosocomial infections [49,56,57]. Our results, linking chronic comorbidities and long-term institutionalisation to high infection rates and poor hospitalisation outcomes, possibly also indicate the need for more comprehensive multimorbidity surveillance programmes and tighter interactions between hospitals and other health facilities for early tackling of complexity and its detrimental consequences.

Our study has multiple limitations. First, we collected data on comorbidity by relying on simplified general pathophysiological categories without considering specific individual diagnoses, which reduces the informativeness of our association analyses. Second, although integrally deployed over one year, our data collection only focused on a single Unit from a university hospital, preventing a comprehensive analysis of different nodes in the healthcare network. In addition, our monocentric design prevented the assessment of healthcare variability across distinct countries and health systems. Third, the number of nurses assisting patients in our ward did not change during the observation timeframe. Therefore, in the absence of a comparator group with a different number of nurses, we were unable to quantitate the extent of correlation between less or more nurses and patient outcomes. Nonetheless, we found a significant association between higher NEMS scores and adverse hospitalisation outcomes, suggesting that nowadays, patient intensity of care in internal medicine departments generates unsustainable nursing workloads exceeding the available resources. Notwithstanding its limitations, this study has its major strengths in its design, assessing three major outcomes for patient care efficiency in a relatively large number of subjects, uncoupling individual factors from unit-related variables and comprehensively following variations in patient status throughout their hospitalisation course.

## 5. Conclusions

In conclusion, our data suggest that patients hospitalised in Internal Medicine Units are exposed to high risks of mortality, prolonged hospitalisation and development of nosocomial infections. These events appear to be significantly affected by the burden of patient active comorbidities and by specific vulnerability factors such as end-stage cancer or upper gastrointestinal disorders but are also strongly impacted by the baseline assistance context in terms of attending physicians during hospitalisation and clinical/social patient environment before and possibly beyond the acute phase. Additional research is needed to identify suitable tools to estimate workforce size for each specific clinical setting. Sustainable planning of healthcare service at the hospital, regional and national level, should, however, not solely be based on financial considerations but also take into account objective measures of workload saturation to optimise individual outcomes and, possibly, global economic and social costs.

## Figures and Tables

**Table 1:** General clinical features.

	All Patients (N = 1073)	Women (n = 445)	Men (n = 628)
**Demographics and outcomes**			
Women: n (%)	445 (41)	445 (100)	0 (0)
Age: median (IQR)	74 (62–82)	73 (61–81)	75 (64–82)
In-hospital deaths: n (%)	119 (11)	48 (11)	71 (11)
Nosocomial infections: n (%)	136 (13)	56 (13)	80 (13)
Time to discharge (days): median (IQR)	12 (8–20)	12 (8–21)	12 (8–19)
**Morbidity**			
ADL/IADL dependence: n (%)	436 (41)	181 (41)	255 (41)
Cardiovascular disorders: n (%)	745 (69)	300 (67)	445 (71)
Cardiac disorders: n (%)	566 (53)	216 (49)	350 (56)
Hypertension: n (%)	583 (54)	242 (54)	341 (54)
Pulmonary disorders: n (%)	517 (48)	205 (46)	312 (50)
Renal disorders: n (%)	362 (34)	134 (30)	228 (36)
Upper gastrointestinal tract disorders: n (%)	103 (10)	41 (9)	62 (10)
Lower gastrointestinal tract disorders: n (%)	105 (10)	51 (11)	54 (9)
Liver disorders: n (%)	170 (16)	62 (14)	108 (17)
Metabolic disorders: n (%)	425 (40)	187 (42)	238 (38)
Immune-mediated disorders: n (%)	180 (17)	110 (25)	70 (11)
Neoplastic disorders: n (%)	350 (33)	132 (30)	218 (35)
End-stage neoplastic disorders: n (%)	76 (7)	33 (7)	43 (7)
Neurological disorders: n (%)	439 (41)	179 (40)	260 (41)
Psychiatric disorders: n (%)	100 (9)	51 (11)	49 (8)
Immunocompromised subjects: n (%)	226 (21)	106 (24)	120 (19)
CIRS total score: median (IQR)	9 (6–13)	9 (5–12)	9 (6–13)
CIRS severity score: median (IQR)	0.7 (0.4–0.9)	0.6 (0.4–0.9)	0.7 (0.5–1.0)
CIRS comorbidity score: median (IQR)	3.0 (2.0–5.0)	3.0 (2.0–5.0)	3.5 (2.0–5.0)
**Intensity of care**			
Continuous vital signs monitoring *: n (%)	1045 (97)	432 (97)	613 (98)
Any oxygen support: n (%)	589 (55)	222 (50)	367 (58)
Nasal cannulas or Venturi’s mask: n (%)	488 (45)	183 (41)	305 (49)
Non-invasive ventilation: n (%)	109 (10)	42 (9)	67 (11)
Intravenous treatments: n (%)	999 (93)	399 (90)	600 (96)
Vasoactive circulation support with one drug at least once: n (%)	17 (2)	7 (2)	10 (2)
Vasoactive circulation support with more than one drug at least once: n (%)	0 (0)	0 (0)	0 (0)
Dialysis at least once: n (%)	14 (1)	3 (1)	11 (2)
At least one in-ward non-standard procedure: n (%)	177 (16)	75 (17)	102 (16)
At least one exit from the ward for other procedures: n (%)	760 (71)	315 (71)	445 (71)
Patients requiring surgery: n (%)	27 (3)	14 (3)	13 (2)
NEMS average score: median (IQR)	18 (16–19)	18 (16–19)	18 (16–19)

* urinary output monitoring for the majority of patients.

**Table 2:** Variations in length of stay by patient groups in the training subset (n = 645).

Variable	Positive		Negative		*p*
	n	Length of Stay (Days)	n	Length of Stay (Days)	
ADL/IADL dependence	230	15 (9–25)	415	11 (8–19)	<0.001
Neurological disorders	245	14 (9–23)	400	12 (8–20)	0.029
Any infection	441	13 (9–22)	204	11 (7–19)	0.002
Any nosocomial infection	81	25 (18–35)	564	11 (8–18)	<0.001
Nosocomial infection, n = 1	72	24 (17–32)			<0.001
Nosocomial infection, n > 1	9	62 (59–89)			<0.001
Nosocomial infection, n = 2	5	59 (32–60)			0.001
Nosocomial infection, n = 3	2	68 (62–63)			0.016
Nosocomial infection, n = 4	2	113 (90–136)			0.014
Admitted from nursing homes or ICU	62	26 (16–49)	583	12 (8–19)	<0.001
Requiring surgery	19	22 (14–42)	626	12 (8–21)	0.001
Any oxygen support	334	14 (9–22)	311	12 (7–19)	0.003
NIMV users	55	16 (10–25)	590	12 (8–21)	0.016

**Table 3:** Generalised linear model for hospitalisation length.

Variable	Coefficient	Standard Error	*p*
**Individual variables**			
ADL/IADL dependency	2.01	0.62	0.001
Neurological disorders	0.17	0.55	0.759
Any infection	1.89	0.51	<0.001
Nosocomial infection, n = 1	5.57	1.46	<0.001
Nosocomial infection, n > 1	25.11	11.65	0.031
Admitted from nursing home or ICU	10.30	1.91	<0.001
Requiring surgery	5.22	2.69	0.053
Any oxygen support	1.85	0.66	0.005
Number of on-standard in-ward procedures	1.11	0.69	0.109
Exits from the Unit	2.67	0.19	<0.001
Average NEMS	−0.13	0.07	0.083
**Unit-related variables**			
Unit average length of hospitalisation	0.09	0.11	0.405
Unit average number of infected patients	4.88	5.25	0.353
Unit average NIMV users	−1.58	5.61	0.778
Patient/physician ratio	0.35	0.10	<0.001
**Intercept**	−3.58	4.37	0.411

## Data Availability

Data supporting this work can be shared upon reasonable requests to the corresponding author.

## Supplementary Materials

The following supporting information can be downloaded at: https://www.mdpi.com/article/10.3390/jpm14010115/s1, Figure S1: patients and variables; Figure S2: patient-related variables; Figure S3: correlation between predicted and real hospitalisation length; Table S1: factors associated with in-hospital mortality in the training subset on univariate analysis (n = 726); Table S2: correlations with length of stay in the training cohort n = 645); Table S3: factors associated with the first nosocomial infection in the training subset on univariate analysis (n = 681).
