# Supplementary material for: Sustainability in Internal Medicine: A Year-Long Ward-Wide Observational Study
Source: J Pers Med. 2024 Jan 20;14(1):115. doi: 10.3390/jpm14010115 (PMC10820757; doi:10.3390/jpm14010115)
Supplement: Supplementary file 1 [file jpm-14-00115-s001.zip › jpm-2810441-supplementary.pdf]

Supplemental material to

Sustainability in internal medicine: a year-long ward-wide observational study

Giuseppe A. Ramirez[1,2,3], Sarah Damanti[2,3], Pier Francesco Caruso[2,3], Francesca Mette[2,3], Gaia Pagliula[2,3], Adriana Cariddi[1,2,3], Silvia Sartorelli[1,2,3], Elisabetta Falbo[2,3], Raffaella Scotti[3], Gaetano Di Terlizzi[3], Lorenzo Dagna[1,2], Luisa Praderio[3], Maria Grazia Sabbadini[2,3], Enrica P. Bozzolo[3], Moreno Tresoldi[3]

1) Unit of Immunology, Rheumatology, Allergy and Rare Diseases, IRCCS Ospedale San Raffaele, Milan, Italy

2) Università Vita-Salute San Raffaele, Milan, Italy

3) Unit of General Medicine and Advanced Care, IRCCS Ospedale San Raffaele, Milan, Italy

Table S1: factors associated with in-hospital mortality in the training subset on univariate analysis (N=726)

Variable	Hazard ratio	95% CI	p
Age (years)	1.04	1.02-1.07	<0.001
ADL/IADL dependance	1.82	1.16-2.87	0.010
Cardiovascular disorder	1.83	1.07-3.13	0.027
Hypertension	1.73	1.09-2.73	0.019
Immune-mediated disorders	0.38	0.17-0.83	0.016
End stage cancer	2.31	1.25-4.27	0.008
Admitted from nursing home residencies or ICU	0.31	0.14-0.70	0.005
Circulation support with at least one drug	8.97	4.09-19.69	<0.001
Average NEMS	1.06	1.02-1.12	0.007
Average total CIRS	1.09	1.05-1.13	<0.001
Average CIRS-SI	3.24	1.92-5.49	<0.001
Average CIRS-CI	1.23	1.11-1.36	<0.001
Number of exits from the Unit	0.82	0.74-0.90	<0.001

Table S2: correlations with length of stay in the training cohort (N=645)

Variable	Rho	p
Individual variables		
Non-standard in-ward procedures	0.240	<0.001
Exits from the Unit	0.239	<0.001
Average NEMS	0.173	<0.001
Number of Non-standard in-ward procedures	0.272	<0.001
Number of Exits from the Unit	0.622	<0.001
Unit-related variables		
Unit average length of hospitalisation	0.079	0.046
Unit number of discharged patients	-0.138	0.001
Unit average infected patients	0.087	0.027
Unit average NIMV users	0.117	0.003
Physician/patient ratio	0.117	0.003

Table S3: factors associated with the first nosocomial infection in the training subset on univariate analysis (N=681)

Variable	Hazard ratio	95% CI	p
Age	1.03	1.02-1.05	<0.001
Cardiovascular disorders	1.73	1.06-2.82	0.029
Hypertension	1.56	1.02-2.42	0.041
Cardiac disorders	1.73	1.13-2.66	0.012
Upper gastrointestinal tract disorders	2.59	1.54-4.35	<0.001
Average total CIRS	1.08	1.04-1.12	<0.001
Average CIRS-SI	2.83	1.60-5.00	<0.001
Average CIRS-CI	1.23	1.10-1.37	<0.001
Average NEMS	1.06	1.01-1.11	0.019
Any oxygen support	1.74	1.10-2.74	0.018
Circulation support with at least one drug	4.67	1.14-19.13	0.032
Number of non-standard in-ward procedures	1.61	1.01-1.33	0.032

Figure S1: patients and variables

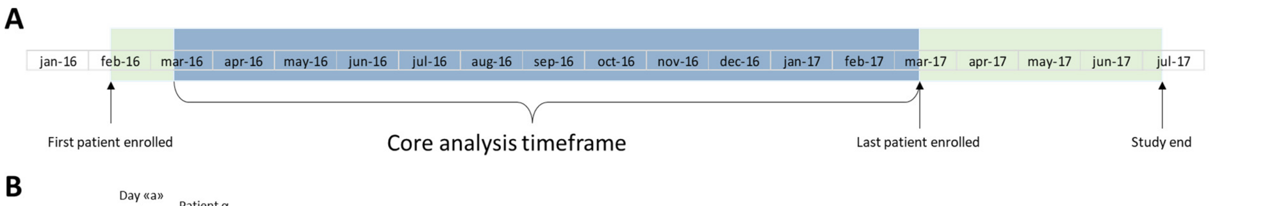

	a	b	c	d	e	f	g	h	i
1	x1aα	x1bζ	x1cζ	x1dζ	x1eζ	x1fζ	x1gζ	x1hζ	x1iζ
2	x2aβ	x2bβ	x2cη	x2dη	x2eη	x2fη	x2gη	x2hη	x2iη
3	x3aγ	x3bγ	x3cγ	x3dγ	x3eγ	x3fγ	x3gγ	x3hι	x3iι
4	x4aδ	x4bδ	x4cδ	x4dδ	x4eδ	x4fδ	x4gδ	x4hδ	x4iκ
5	x5aε	x5bε	x5cθ	x5dθ	x5eθ	x5fθ	x5gθ	x5hθ	x5iλ
...									

γ		ζ		η		θ		κ	
3a-g		1b-i		2c-i		5c-h		4i	
Mean	Worst	Mean	Worst	Mean	Worst	Mean	Worst	Mean	Worst

W(a)		W(b)		W(c)		W(d)		W(e)		W(f)		W(g)		W(h)		W(i)	
aαβγδε		bζβγδε		cζηγδθ		dζηγδθ		eζηγδθ		fζηγδθ		gζηγδθ		hζηιδθ		iζηικλ	
Mean	Worst	Mean	Worst	Mean	Worst	Mean	Worst	Mean	Worst	Mean	Worst	Mean	Worst	Mean	Worst	Mean	Worst

γ		ζ		η		θ		κ	
W(a-g)		W(b-i)		W(c-i)		W(c-h)		W(i)	
Mean	Worst	Mean	Worst	Mean	Worst	Mean	Worst	Mean	Worst

This figure shows the main methodological principles supporting data collection for this work. Panel A depicts the total duration of the study (green) and the core timeframe for data collection (blue). In panel B a schematic representation of the algorithm to obtain a set of derived variables from a given "x" source variable is provided. In this set of graphs, patients are labelled with Greek letters, days by Latin letters and beds by numbers. Grey cells refer to a subset of subjects (γ, ζ, η, θ, κ) to be analysed for variable "x", white cells to patients being admitted before or after subject γ, ζ, η, θ, κ (α,β,δ,ε,ι,λ) and excluded from analysis. For any x variable, individual patient data were collected day by day (legend in the upper left corner). The mean and worst "x" scores were also calculated ward-wide on a daily basis, by integrating data from all inpatients in a given day (lower left sub-table). Intraindividual variations in "x" values were captured by mean and worst individual "x" throughout each patient specific hospitalisation interval (upper right sub-table). The mean and worst "x" values of the whole unit were also differentially calculated for each subject according to each subject's individual hospitalisation timeframe (lower right sub-table).

Figure S2: patient-related variables

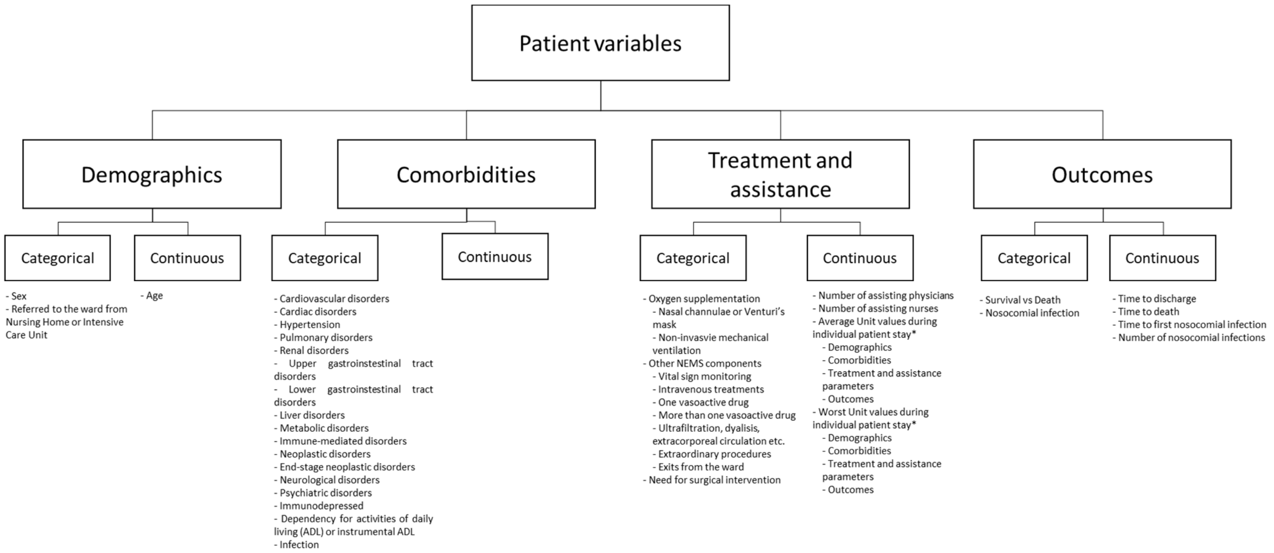

Schematic diagram showing a breakdown of patient-related variables by topic (demographics, comorbidities, treatment and assistance, outcomes) and type (categorical vs continuous variables). * Please refer to figure S1 and the main text for the calculation of patient-referred Unit average and worst values.

Figure S3: correlation between predicted and real hospitalisation length

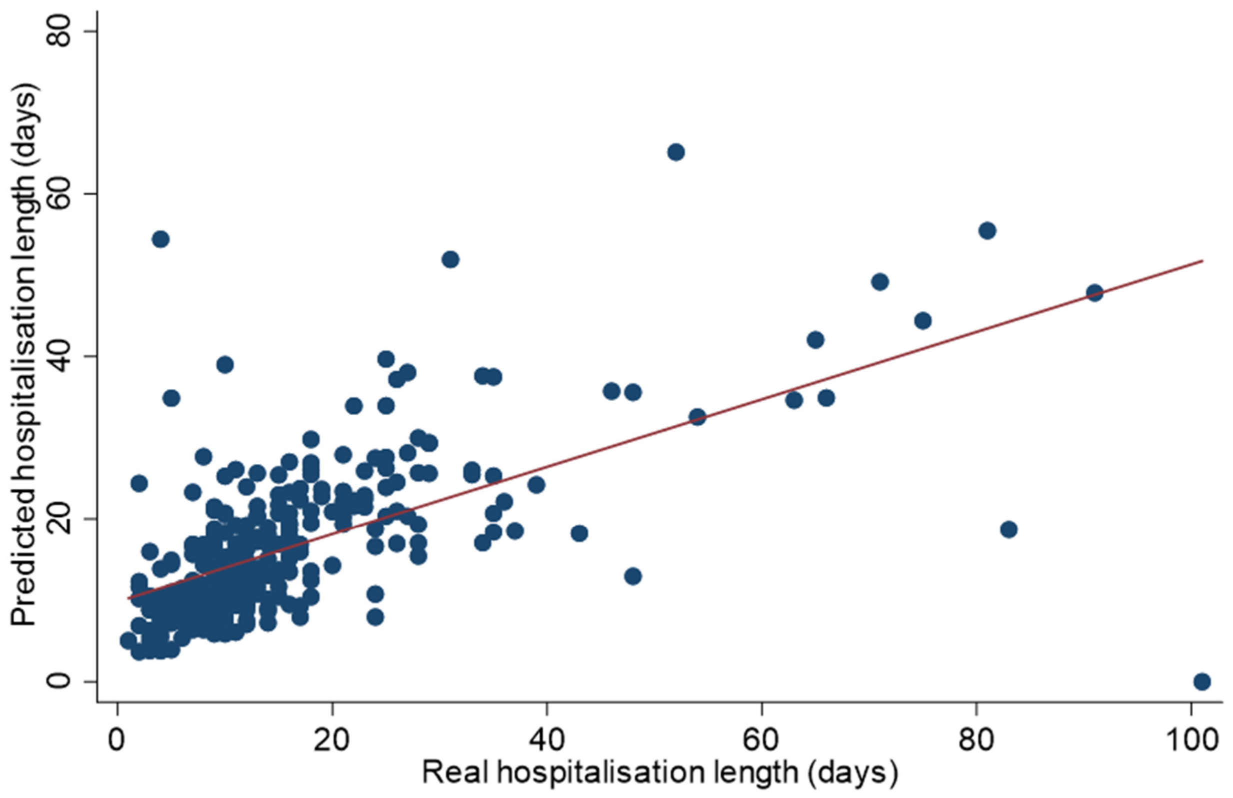

This graph shows the relation among predicted lengths of hospitalisation calculated in the validation subset based on multivariate analysis in the training subset and measured hospitalisation lengths in the same subset.
